# Barriers to Yearly Diabetic Retinopathy Screening Attendance: A Retrospective Clinical Audit

**DOI:** 10.7759/cureus.75474

**Published:** 2024-12-10

**Authors:** Upamanyu Leo Chanda, Anshuman Madasu, Ananya Bhardwaj, Mohamed Mohyudin

**Affiliations:** 1 Emergency, New Cross Hospital, Royal Wolverhampton Trust, Wolverhampton, GBR; 2 Ophthalmology, Calderdale Royal Hospital, Halifax, GBR; 3 Cardiology, Harrogate District Hospital, Harrogate, GBR

**Keywords:** clinical audit, diabetic retinopathy, eye screening, nice guidelines, quality improvement projects

## Abstract

Diabetic retinopathy is the most common sight-threatening complication of diabetes, necessitating regular monitoring of progression via diabetic eye screening (DES). The National Institute for Health and Care Excellence (NICE) recommends DES annually for diabetic patients aged 12 years and older. This retrospective clinical audit assessed the reasons behind non-attendance and evaluated the adherence to guidelines set by NICE in a general practice with approximately 9000 patients.

The *SystmOne *patient database was used to identify patients who had missed DES in the last 15 and 36 months, with the latter being categorised as ‘repeat non-attenders.’ A survey of every third patient who missed screening in the last 15 months highlighted mental health issues, lack of DES awareness, and transport difficulties as primary reasons for non-attendance. The DES uptake rates recorded at the practice were 77.2% (n=465) for the 15-month cohort and 83.6% (n=503) for the 36-month cohort, exceeding the NHS DES target of 75%.

Proposed interventions to increase attendance to DES include telephone prompts for patients with mental health concerns, text message reminders, and online educational tools to improve uptake. Despite standards exceeding the national guidelines, this audit demonstrates the importance of addressing specific barriers to enhance screening rates, potentially increasing the detection of early retinopathy and improving patient outcomes.

## Introduction

Diabetic retinopathy (DRP) is the most common microvascular complication in diabetic patients, with a prevalence of 54.6% in patients with type 1 diabetes and 30.0% in patients with type 2 diabetes [1]. It is also a leading cause of blindness and low vision amongst persons of working age in developed countries [2]. In the initial stages of non-proliferative diabetic retinopathy, most patients do not notice any visual changes, as these can take years to develop. Regular attendance at diabetic eye screening (DES) is therefore vital to identify potentially sight-threatening diabetic retinal changes and slow the progression of DRP.

The National Institute for Health and Care Excellence (NICE) guidelines recommend annual screening for DRP for all persons with diabetes over the age of 12 years [3]. During DES, two standard digital fundus photographs are taken from each eye, and the images are subsequently graded according to the English National Screening Programme for Diabetic Retinopathy (ENSPDR) classification system. Individuals classified as having “sight-threatening retinopathy” are referred to ophthalmology clinics for assessment. Current guidelines in primary care recommend that general practitioners promptly identify newly diagnosed diabetic patients and refer them for DES, which is to be completed within three months of referral, followed by annual screenings [4]. The Public Health England-commissioned General Practice to Diabetic Retinopathy Screening (GP2DRS) programme electronically transfers data from GP diabetes registers directly to eye screening services, ensuring automatic identification of individuals needing regular screening [5]. As of 2022, the NHS DES programme invites eligible patients for annual screening, with a minimum uptake target of 75%, aiming for over 85% [6]. Patients requiring closer monitoring follow surveillance pathways with recall intervals of one, three, six, nine, or 12 months.

Aims

The primary aim of this retrospective audit was to assess whether patients at a general practice adhered to NICE guidelines for DRP screening. Additionally, the audit explored why some patients fail to engage with screening programs by identifying factors influencing non-attendance.

## Materials and methods

This single-centre retrospective clinical audit was conducted at a medium-sized general practice, The Park Surgery (General Practice), East Midlands, United Kingdom, with approximately 9000 patients. The audit aimed to assess adherence to DES guidelines through the identification of individuals on the diabetes register who had not attended DES appointments over specific time frames. The *SystmOne* patient database, an electronic health record system used widely across NHS practices, was used to identify individuals on the diabetes register that did not attend DES in the last 15 months and the last 36 months prior to 28th September 2022. The purpose of both time frames was in order to assess the trends in non-attendance over short-term and extended periods.

The upper time limit of non-attendance was recorded at 36 months, as individuals that do not attend DES for a period of at least three annual cycles are classed as “repeat non-attenders” by Public Health England DES guidelines [7]. This category of individuals is of high clinical significance, as repeat non-attenders are more likely to exhibit delayed presentations of proliferative retinal changes. Through use of a 36-month upper limit, this audit specifically targeted patients at highest risk due to extended screening absence, thus providing greater insight into long-term trends in adherence. Individuals below the age of 12 years were excluded from the study, in line with NICE guidelines.

Every third patient who had missed screening in the past 15 months was contacted and asked a series of questions (Appendix 1) to explore their reasons for non-attendance. These individuals were contacted by telephone in order to identify possible barriers to attendance, including logistical issues, personal health beliefs, and a lack of understanding regarding the importance of retinal screening in diabetes. The utilization of this structured questionnaire allowed for qualitative insight into patient-specific factors that may have contributed to non-attendance, therefore supporting the development of targeted interventions to increase adherence to DES in this demographic.

## Results

Of the 602 individuals on the diabetes register, 137 did not attend DES within the last 15 months (77.2% uptake), 99 out of 602 individuals did not attend DES within the last 36 months (83.6% uptake), and 38 out of 602 individuals attended DES within the last 15 months but did not attend their yearly screening for the two consecutive previous years before their last appointment. The mean age of non-attenders within the 15-month cohort was 60.6 years, ranging from 12 to 98 years. Additionally, this cohort had a higher proportion of male non-attenders (58.4%, n=80) than females (41.6%, n=57). 

**Figure 1:**
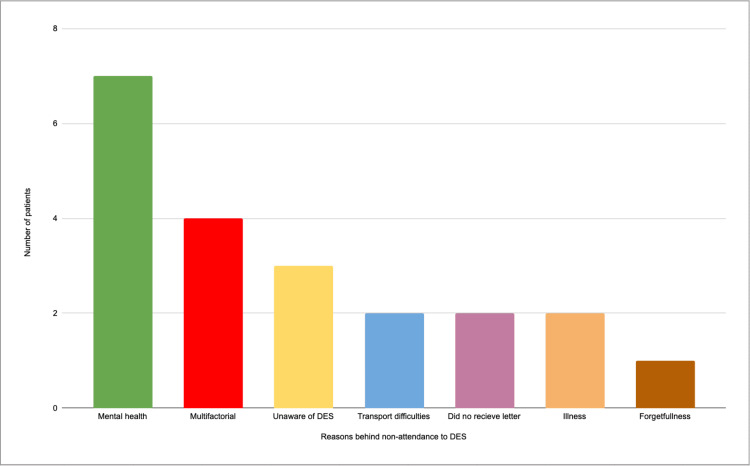
Chart showing the responses to question 5 of the questionnaire. DES: diabetic eye screening

A total of 45 patients were included in the telephone survey, with 21 agreeing to participate and 24 not responding. All participants had attended DES in the past. The most common reasons for non-attendance are outlined in Figure 1. The leading reason for non-attendance to DES appointments was issues related to mental health (n=7), such as depression, anxiety, and paranoia.

The second most common reason for non-attendance was multifactorial, with participants citing a mix of factors such as busy lifestyles, lack of current visual issues, and low perceived importance of DES. These individuals reported difficulty taking time off work and managing inflexible schedules. Notably, none of these factors were cited as the sole reason for missing appointments, reinforcing the multifaceted nature of non-attendance. Other reasons included a lack of awareness about DES (n=3), transport difficulties (n=2), not receiving the reminder letter (n=2), illness (n=2), and forgetfulness (n=1). 

## Discussion

This particular general practice demonstrated a higher rate of DES uptake among the 15-month cohort and the 36-month cohort (77.2% and 83.6%, respectively) than the minimum aim (75%) outlined in the 2022 NHS DES programme, and the 36-month cohort had a higher rate of DES uptake than both the regional and national data. In contrast, the 15-month cohort studied in this audit displayed a lower rate of DES uptake when compared to the overall data for the Derbyshire Diabetic Eye Screening programme (81.0%) and the national data (82.6%) [8].

The findings from this study indicate that the most prominent patient-specific factors contributing to non-attendance for diabetic eye screening (DES) were mental health issues (33.3%, n=7), lack of DES awareness (14.3%, n=3), and transport difficulties (9.5%, n=2). These results align with existing literature, although some differences are noted. For instance, Dantas et al.'s [9] systematic review highlights non-attendance as more prevalent among younger adults and individuals from lower socioeconomic backgrounds, trends observed across multiple medical specialities [10]. Although our study did not specifically investigate socioeconomic factors, other research has identified mental health problems in young adults as a significant predictor of missed appointments in primary care [11]. While mental health issues were noted as a barrier to attendance in our study, illness was more frequently cited [12,13]. However, since the specific nature of the illnesses was not detailed, it is possible that mental health issues contributed to some of these cases, though the available data does not provide sufficient evidence to confirm this.

The 2019 study by Piyasena et al. [14] illustrates a lack of knowledge regarding diabetic retinopathy as the most consistent barrier to DES. Reduced awareness of diabetic retinopathy as a barrier to DES has been documented from as early as 2011 by van Eijk et al. [15] and is partially supported by our findings, as unawareness of diabetic retinopathy and DES was the second most frequent response. Of note, both previously mentioned studies identify fear as an identified barrier to DES attendance. However, none of the individuals contacted in our study described fear of eye screening as a factor contributing to non-attendance, despite 100% of respondents having participated in DES at some point in the past.

Prothero et al. [16] and Graham-Rowe et al. [17] both identified similar barriers in their studies, particularly limited knowledge of DES's importance and environmental factors like appointment flexibility. Graham-Rowe et al. systematic review of barriers and enablers to diabetic retinopathy screening (DRS) also highlighted competing time demands due to work and family responsibilities, compounded by a lack of appointment flexibility [17]. Our findings are in line with these, as professional engagements and busy lifestyles were frequently cited by participants in our study as reasons for non-attendance. Both studies suggest that practical issues, rather than solely a lack of awareness, are key contributors to missed appointments for DES.

Administrative and communication issues were not a significant factor in our study, possibly due to the efficiency of the reminder system used by this general practice. In contrast, previous studies [18,19] have reported that inefficient hospital administration can account for a significant portion of non-attendance. 

Transportation problems were also a recurring theme in our study, consistent with existing literature on barriers to healthcare access for diabetic patients [13]. Geographic access, poor public transport options, and increased commuting distances were all cited as significant obstacles. These issues align with findings from other studies that highlight transport difficulties as a key barrier to accessing routine healthcare services.

Individuals who described their current lack of visual issues as a factor were reminded of the progressive nature of diabetic retinopathy and were directed towards the key information and statistics on sight loss in the UK document produced by the Royal National Institute of Blind People (RNIB) [20]. For the individuals who were unaware about retinal screening and the potentially sight-threatening nature of advanced diabetic retinopathy, a brief explanation regarding diabetic retinopathy as a complication of diabetes and recommendations from the NHS website and a document by the Diabetes Research and Wellness Foundation [21] were offered. 

Recommendations

The two most prevalent barriers to DES attendance were identified as mental health and knowledge/awareness about diabetic retinopathy and the screening programme. Firstly, addressing the underlying mental health of these patients may be the most important first step, as ensuring that the patient is receiving appropriate support for their condition may increase the likelihood that they engage in health education and thus improve uptake for yearly retinal screening. For individuals who require extra support, a possible intervention could be the addition of a telephone prompt 48 hours before their screening appointment. A short telephone conversation with these individuals explaining the process of attending the screening services and providing gentle encouragement shortly prior to their appointments may improve uptake in this category. It is important to ensure that patients are encouraged rather than forced, as some may see this early contact from the clinic as intrusive or anxiety-inducing. A limitation of this method would be a high rate of unanswered calls due to patients being at work or otherwise preoccupied, alongside an increased workload for the practice.

An alternative intervention for individuals in the other categories or who prefer to opt out of phone call reminders could be the implementation of a text message reminder system. A text message could be sent to applicable individuals alongside the invitation to the DES letter, thus reducing the likelihood of missing the initial letter and subsequently improving uptake. Furthermore, the use of a text message reminder system would allow for the addition of appropriate website links, infographics, and leaflets to inform or remind patients regarding the importance of DES and the risks of missing annual screening, while simultaneously providing a less anxiety-inducing platform for individuals that may be sensitive to discussion over the telephone.

As highlighted by similar studies, addressing the lack of knowledge and awareness surrounding diabetic retinopathy and sight loss as a complication of diabetes is a crucial factor in improving the overall uptake of DES. A possible intervention could be the implementation of an online patient education system accessible to all individuals on the diabetes register. A short self-study e-learning tool explaining the background, risk factors, and complications of diabetic retinopathy may aid patients by increasing their understanding of the ocular manifestations of diabetes and preventing the progression of retinal disease. A 2018 study by Beaser et al. [22] illustrated that patient-focused education can empower patients with long-standing diabetes and encourage them to engage with both primary care and their eye services. This method may be more effective than the classical route of providing leaflets, as a short online self-study programme utilising active recall may improve knowledge retention. The downside to an online education system would be ensuring that patients actively engage with the programme, as some individuals may have a negative perception of this tool as being akin to “homework” and difficult to make time for in an already busy schedule.

Limitations

The main limitation of this study was the small sample size, as 53.3% (n=24) of individuals who were called for the questionnaire did not answer the phone. Factors that may have contributed to reduced pickup rates may include the fact that the calls were made during normal working hours and that the patients were not expecting a telephone call on this day from the general practice.

In order to improve this study in the future, a larger sample of patients could be called, if not the entire list of individuals on the non-attender list. Furthermore, due to the subjective nature of the questionnaire, it was impossible to list every reason for non-attendance, particularly in individuals who had multiple reasons, thus requiring the “multifactorial” category. For future projects, it would be beneficial to utilise more close-ended questions in order to improve the consistency of the responses.

## Conclusions

This retrospective audit highlights several key factors influencing non-attendance to DES at a general practice, demonstrating the importance of addressing barriers to engagement in order to improve uptake. The most prevalent reasons for non-attendance were linked to mental health issues and a lack of knowledge about diabetic retinopathy. Implementing targeted interventions, such as phone call prompts for patients with known mental health conditions, text message reminders, and online educational tools, may effectively address these barriers and encourage participation. Despite achieving a higher DES uptake than the minimum target set by the NHS DES programme, this audit emphasises the need for continuous efforts to raise awareness and support patients in attending regular screening. Future re-audits following the implementation of the proposed interventions could help evaluate the impact of these strategies and provide further insights into optimising diabetic retinopathy screening in primary care.

## Appendices

Appendix 1 

**Table 1:** Questionnaire for sample cohort and response type

Question Number	Question	Response Type
1	Have you received a letter in the last 15 months inviting you for diabetic eye screening?	Yes/No
2	Have you had diabetic eye screening in the past?	Yes/No
3	Are you aware of why diabetic eye screening is conducted, and the impact of diabetes on the eye?	Yes/No
4	Did you have any travel/transport difficulties accessing eye screening services?	Short answer (free text)
5	What were your main reasons for non-attendance?	Short answer (free text)
